# *Cladophialophora inabaensis* sp. nov., a New Species among the Dark Septate Endophytes from a Secondary Forest in Tottori, Japan

**DOI:** 10.1264/jsme2.ME16016

**Published:** 2016-06-03

**Authors:** Erika Usui, Yusuke Takashima, Kazuhiko Narisawa

**Affiliations:** 1 United Graduate School of Agricultural Science, Tokyo University of Agriculture and TechnologySaiwaicho 3–5–8, Fuchu, Tokyo 183–8509Japan; 2 Department of Bioresource Science, College of Agriculture, Ibaraki University3–21–1 Chuoh, Ami, Inashiki, Ibaraki 300–0393Japan

**Keywords:** *Cladophialophora* sp., taxonomy, root endophyte

## Abstract

A novel species of *Cladophialophora* is herein described from the natural environment of secondary forest soil in Japan, which was able to be colonized by the host plant root. Morphological observations indicated that the isolate is distinct from previously identified species, and, thus, is described as the new species, *C. inabaensis* sp. nov.

The genus *Cladophialophora* comprises dematiaceous hyphomycetes that produce simple conidiophores, often reduced to conidiogenous cells with catenate ellipsoidal to fusiform conidia, formed in branched or unbranched acropetal chains (1, 13). This genus was initially created to accommodate species exhibiting *Phialophora*-like conidiogenous cells in addition to conidial chains (2). This fungus has been isolated from diverse environmental sources such as dead plant material, rotten wood, and soil (1, 2, 5–9, 11, 16, 19). *Heteroconium chaetospira* (≡*Cladophialophora chaetospira*) is a well-known dark septate endophytic (DSE) fungus (26).

DSE fungi are defined as conidial or sterile ascomycetous fungi that colonize living plant root tissues intracellularly and intercellularly without causing any apparent negative effects, such as tissue disorganization, or forming any typical mycorrhizal structures (15). These fungi are ubiquitous colonizers of the non-mycorrhizal roots of many plant species in the natural ecosystem, particularly under cool and nutrient-stressed conditions. DSE associations have been identified in approx. 600 plant species of 320 genera in 114 families, including non-mycorrhizal plant species (14, 20), and may benefit their host plants by facilitating the uptake of plant mineral nutrients, including phosphorus and nitrogen (26). In spite of their widespread occurrence, limited information is available on the ecological behavior or existence of novel DSE species, particularly in temperate areas.

In the present study, an apparently undescribed species with *Cladophialophora* was successfully isolated from temperate secondary forest soil using baiting plant roots. This information is necessary in order to show the diversity of this fungal group and gain a better understanding of its nature.

The isolates used in the present study were derived from the culture collection of the laboratory of Microbial Ecology, Ibaraki University. The isolate EUCL1 (MAFF 245257), obtained from eggplant roots in 2010, showed dark septate mycelia. Stock cultures were maintained on petri dishes of 50% cornmeal malt yeast (CMMY) agar medium and oatmeal (OM) agar at 23°C.

A pure fungal culture was grown at room temperature on 50-mm Petri dishes containing OM agar. Small pieces (approximately 2×2×2 mm) of Pablum agar (Mead Johnson mixed Pablum; Canadian Post Corporation, Ontario, Canada, 25 g; Bacto agar, 5 g; ultrapure water, 250 mL) were sandwiched between two 18×18-mm cover glasses (Matsunami Glass, Osaka, Japan), placed in a 9-cm water agar plate to provide humidity, and then incubated at room temperature. After 2 to 4 weeks, when the culture had grown sufficiently, Pablum agar was carefully removed and the cover glasses were appropriately mounted on 76×26 mm micro slide glasses using PVLG (Polyvinyl alcohol, 16.6 g; lactic acid [Wako Chemical, Osaka, Japan], 100 mL; glycerin [Wako Chemical], 10 mL; ultrapure water, 100 mL) mounting medium. Conidiogenous cells and conidia were observed under a light microscope (BX51; Olympus, Tokyo, Japan). More than one hundred conidia were measured.

The isolate EUCL1, obtained from eggplant roots, showed dark septate mycelia and grew slowly on PDA medium at room temperature. After three weeks, colonies reached 24.8 mm in diameter and were dark gray to dark brown in color, felty, with lanose aerial mycelia (Fig. 1A). The optimal growth of the fungus was between 23 to 30°C (Fig. 2). Sterile hyphae were branched, septate, smooth, typically 1.6 to 3.6 μm in width, and subhyaline to brown. Differentiated conidiophores were absent or only rarely present and semi-macronematous. Variations were also observed in conidial sizes, which ranged between 3.4 and 7.2 μm, with an average of 4.6 μm in length, and between 3.0 and 4.9 μm, with an average of 3.9 μm in width. The newly isolated fungus was morphologically characterized by slow-growing melanized hyphae that formed semi-macronematous conidiophores producing coherent conidial chains. Thus, this isolate was considered to be a member of the genus *Cladophialophora*. The type species of *Cladophialophora*, *C. carrionii*, is one of the most frequent etiological agents of human chromoblastomycosis in semi-arid climates (8, 25), and the conidia of *C. carrionii* are mostly limoniform to short fusiform with a length:width ratio of less than 4:1 (7); whereas conidia of this isolate are subglobose with a length:width ratio of 1 to 2:1. This new isolate morphologically resembles *C. boppii* in respect of conidia that are subglobose and in a simple chain; however, *C. boppii* sometimes produces larger intercalary or terminal cells that are ellipsoid and dictyoseptated, and measure up to 14 μm in diameter (4). Conidia of *C. parmeliae*, isolated from lichen host tissues, mainly have 1 septate (9). Moreover, Obase *et al.* (2016) recently reported a new species of *Cladophialophora* isolated from the sclerotia of *Cenococcum geophilum* in USA. *C. floridana* and *C. tortuosa* also form subglobose to oblong conidia, whereas the conidia of *C. tortuosa* are more frequently and distinctly phaseoliform or sigmoid in shape. Only *C. floridana* produces conidia-like cells that appear to be larger than conidia (18). Thus, this isolate is morphologically distinct from all previously described species of *Cladophialophora*.

Genomic DNA was extracted from the mycelia of this isolate grown on OM medium using PrepMan^TM^ Ultra Sample Preparation Reagent (Applied Biosystems, CA, USA). We amplified the internal transcribed spacer (ITS) 1-5.8S-ITS2 regions and partial 28S large subunit (LSU) region of the rRNA gene using Ex Taq DNA polymerase (Takara, Otsu, Japan) with the fungal-specific primer ITS1F (12) and universal primer LR5 (27). Amplification was performed using Takara PCR Thermal Cycler Dice (Takara Bio model TP 600) under the following conditions: at 95°C for 5 min, followed by 30 cycles at 95°C (30 s), 54°C (30 s), and 72°C (90 s), and then at 72°C for 10 min. PCR products were purified by polyethylene glycol and ethanol precipitation. The cycle sequence reaction was then conducted with a BigDye Terminator Cycle Sequencing Ready Reaction Kit (Applied Biosystems) using the primers ITS1F, ITS3 (28), LR0R (5′-ACCCGCTGAACTTAAGC-3′) (Vilgalys mycology lab website, http://sites.biology.duke.edu/fungi/mycolab/primers.htm), and LR5 according to the manufacturer’s instructions and was sequenced on an Applied Biosystems 3130xl genetic analyzer (Applied Biosystems). The partial sequences obtained from each sequencing primer were assembled to a contig using GeneStudio Professional software version 2.2.0.0 (www.genestudio.com), and the sequence (1,424 positions) was deposited under the accession number LC128795 in the DDBJ.

Prior to a phylogenetic analysis of the isolate EUCL1, the ITS1-5.8S-ITS2 region, partial LSU region, and the ITS1-5.8S-ITS2 and LSU region concatenated sequences were identified through a BLAST-N search in the NCBI. Each query contained 533, 891, and 1,424 positions, respectively.

The results of the BLAST search revealed that the sequence of the ITS1-5.8S-ITS2 region of the isolate EUCL1 showed low similarities (89 to 90%) to *C. parmeliae* CBS 129337 (JQ342180), *C. chaetospira* CBS 514.63 (KF928449), and *C. carrionii* CBS 160.54 (KF928453). The sequence of the partial LSU region of the isolate EUCL1 showed high similarities (98 to 99%) to *Phialophora americana* MUCL 40613 (AF050280), *C. tortuosa* BA4b006 (AB986424), and *P. verrucosa* IFM 41871 (AB550778). A comparison of the results of individual regions showed that the ITS1-5.8S-ITS2 and LSU region concatenated sequence of the isolate EUCL1 displayed moderate similarities (95%) to *C. carrionii* CBS 160.54 (AF050262), *C. floridana* SR3028 (AB986343), and *C. chaetospira* CBS 514.63 (EU035406). These results demonstrated that the phylogenetic group we handled was polyphyletic and had different evolutionary patterns for each region.

In the phylogenetic analysis, we retrieved sequences from GenBank as shown in Table 1. Since, in some taxa that we included in the analysis, the deposited sequences of the ITS1-5.8S-ITS2 region and LSU region were separated, we aligned each region separately. Sequences containing at least a partial ITS1-5.8S-ITS2 region were partitioned into ITS1, 5.8S, ITS2, and partial LSU regions using ITSx version 1.0.11 (3), and the sequences containing only a partial LSU sequence (Table 1) were then added to the alignment of the partial LSU region generated by ITSx. Each partitioned region was individually aligned with the MUSCLE (10) implemented with MEGA version 6.06 (22), and ambiguous regions of both sides in each alignment were deleted. As a result, the alignment block of each region contained 174, 161, 201, and 505 positions, respectively. Since the ITS1-5.8S-ITS2 region and LSU region of this phylogenetic group have different evolutionary patterns, we compared three partition models, *i. e.*, separate, partitioned equal mean rate, and non-partitioned to a concatenated dataset (ITS1, 5.8S, ITS2, and partial LSU regions: a total of 1,041 positions) by Kakusan4 (24) with a default setting except for considering the non-partitioning of loci. For the phylogenetic analysis, RAxML version 8.1.5 (21) was used. As a result, the separate partition model was selected by the lowest AIC in Kakusan4. Maximum likelihood trees were reconstructed by RAxML version 8.1.5 with the separate partition model selected by Kakusan4, GTR+G as a substitution model, and a bootstrap analysis with 1,000 replicates. The bootstrap values for each node were mapped by the pgsumtree command in Phylogears2 (23) for the reconstructed best-fit tree. The ML tree showed that the phylogenetic position of EUCL1 was different from the other *Cladophialophora* spp. (Fig. 3) and closely related taxa identified by the BLAST search. In order to accommodate this isolate, the following new species was proposed: *C. inabaensis* E. Usui, & K. Narisawa, sp. nov.

The new species, *C. inabaensis*, morphologically resembles *C. boppii*, which has only rarely been reported as the cause of cutaneous infection and is an agent of subcutaneous phaeohyphomycosis in humans (17). In contrast, *C. inabaensis* was isolated from eggplant roots using the baiting method. This dematiaceous fungus colonizes the inner cortical tissue of Chinese cabbage roots without causing any apparent pathogenic symptoms or forming any mycorrhizal structures, and is suggested to be a DSE fungus. Several studies have reported on plant-associated *Cladophialophora* species such as *C. hostae*, *C. scillae*, *C. proteae*, and *C. sylvestris* (6). *H. chaetospira* (≡*C. chaetospira*) is a well-known DSE fungus. Previous studies showed that *H. chaetospira* colonizes the roots of Chinese cabbage, and transfers nitrogen (26). The new species, *C. inabaensis*, also showed endophytic features in the inoculation tests to host plants; however, the precise ecological niches are still unknown. Additional plant inoculation tests on *C. inabaensis* with different plant species under various environmental conditions are still needed.

## Taxonomy

**Fig. 1:**
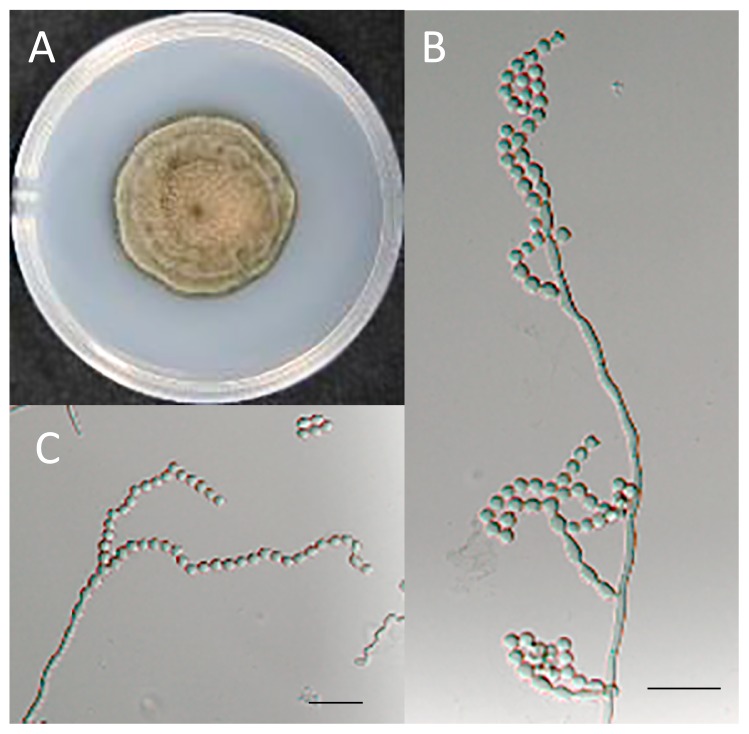
*Cladophialophora inabaensis* (MAFF245257). (A) Culture on PDA after a 3-week incubation at 23°C. (B) Conidiophores laterally or terminally on undifferentiated hyphae. (C) Long, strongly coherent conidial chains. Scale bar=20 μm.

*Cladophialophora inabaensis* E. Usui, & K. Narisawa, sp. nov. (Fig. 1)

MycoBank no.: MB815832.

**Fig. 2:**
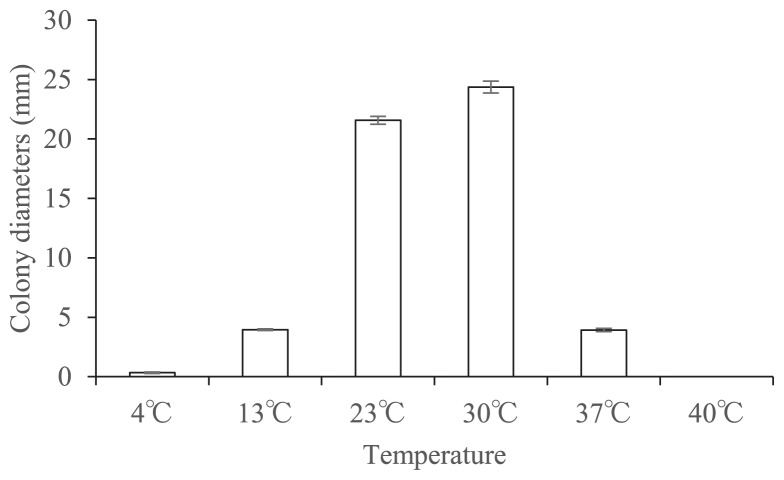
Colony diameters at various temperatures ranging between 4°C and 40°C, measured after 21 d on PDA, were calculated for *Cladophialophora inabaensis* (MAFF245257).

Mycelial hyphae hyaline to brown, irregularly septate, branched, 1.6–3.6 μm wide. A novel species form, non-septate, melanized, subglobose (length:breadth ratio <1 to 2:1) conidia produced in coherent and infrequently branched chains that often arise from semi macronematous differentiated conidiophores. Conidiophores producing up to more than 10 conidia, releasing the conidia schizolytically. Conidia one-celled, brown to dark brown in color, smooth-walled. There were also variations in the size of these conidia 3.4–7.2×3.0–4.9 μm (Fig. 1B and C). Chlamydospores absent. Phialides absent. Teleomorph unknown. Optimal growth at 23 to 30°C, and was still able to grow at 37°C. No growth at 40°C (Fig. 2).

Etymology: inabaensis in reference to the old Japanese local administrative division from which the fungus was isolated.

Ex-holotype: Isolate EUCL1 (MAFF245257) obtained from a secondary forest soil in Tottori, Japan in 2010.

Distribution: only at the type location in Tottori.

## Figures and Tables

**Fig. 3:**
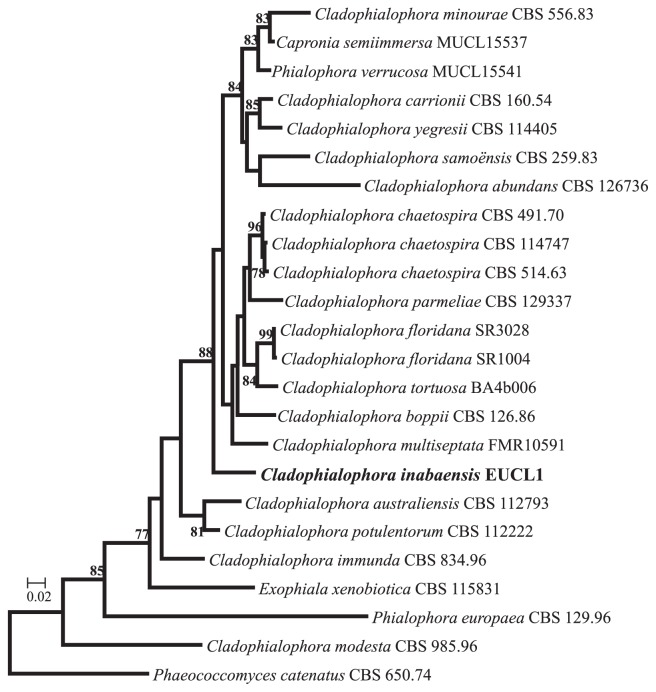
Maximum likelihood phylogenetic tree on a concatenated dataset of ITS1, 5.8S, ITS2, and partial LSU regions (1,041 positions) using RAxML version 8.1.5 with the separate partition model selected by Kakusan4, GTR+G as a substitution model (log likelihood= –5741.841371), and a bootstrap analysis with 1,000 replicates. Bootstrap values >70% are shown at nodes. *Phaeococcomyces catenatus* CBS 650.76 was used as an outgroup.

**Table 1:** Fungal isolates used for the phylogenetic analysis in this study.

Taxon	Strain	GenBank accessions		

		ITS+LSU	ITS	LSU
*Capronia semiimmersa* (≡*Phialophora americana*)	MUCL15537	AF050283	—	—
*Cladophialophora abundans*	CBS 126736 T	—	KC776592	KC812100
*Cladophialophora australiensis*	CBS 112793 T	EU035402	—	—
*Cladophialophora boppii*	CBS 126.86 T	—	EU103997	FJ358233
*Cladophialophora carrionii*	CBS 160.54 T	AF050262	—	—
*Cladophialophora chaetospira*	CBS 114747	EU035403	—	—
*Cladophialophora chaetospira*	CBS 491.70	EU035405	—	—
*Cladophialophora chaetospira*	CBS 514.63	EU035406	—	—
*Cladophialophora floridana*	SR1004	AB986344	—	—
*Cladophialophora floridana*	SR3028 T	AB986343	—	—
*Cladophialophora immunda*	CBS 834.96 T	—	EU137318	KC809990
*Cladophialophora inabaensis*	EUCL1 T	LC128795	—	—
*Cladophialophora minourae*	CBS 556.83 T	AY251087	—	—
*Cladophialophora modesta*	CBS 985.96	—	NR_121459	KF928485
*Cladophialophora multiseptata*	FMR10591 T	—	HG003668	HG003671
*Cladophialophora parmeliae*	CBS 129337	JQ342180	—	—
*Cladophialophora potulentorum*	CBS 112222	EU035409	—	—
*Cladophialophora samoënsis*	CBS 259.83 T	—	EU137291	KC809992
*Cladophialophora tortuosa*	BA4b006 T	AB986424	—	—
*Cladophialophora yegresii*	CBS 114405 T	—	EU137322	KC809994
*Exophiala xenobiotica*	CBS 115831	—	AY857539	FJ358246
*Phaeococcomyces catenatus*	CBS 650.76 T	AF050277	—	—
*Phialophora europaea*	CBS 129.96	—	JQ766440	JQ766487
*Phialophora verrucosa*	CBS 839.69	EU514701	—	—
